# Kefir and healthy aging: revealing thematic gaps through AI-assisted screening and semantic evidence mapping

**DOI:** 10.3389/fragi.2025.1628474

**Published:** 2025-10-02

**Authors:** Francesco Chiani

**Affiliations:** Institute of Biochemistry and Cell Biology, IBBC, CNR, Monterotondo, Rome, Italy

**Keywords:** kefir, probiotcs, aging, systematic evidence mapping, microbiota, gut-brain axes, multisystem health effects, systematic review

## Abstract

Kefir, fermented milk rich in probiotics, has attracted growing attention for its potential anti-aging effects. Yet, studies specifically addressing kefir in the context of aging remain limited and scattered across diverse biological fields. To overcome this fragmentation, we applied an integrative approach that combines a cutting-edge AI-assisted algorithm for evidence screening with a Python-based semantic clustering pipeline. This allowed us to systematically map and classify the existing literature into four functional domains of aging: changes in body composition, energy balance, homeostatic signaling networks, and neurodegeneration. The resulting evidence map revealed a marked thematic imbalance, with most studies concentrated in mechanistic pathways such as inflammation and oxidative stress, and far fewer addressing neurocognitive or metabolic outcomes. This asymmetry suggests a structural bias in current research priorities and highlights the need to expand kefir-related studies toward more clinically relevant aging endpoints. By merging AI with domain-specific linguistic tools, our study provides a reproducible and data-driven strategy to uncover thematic blind spots and guide future investigations into kefir’s anti-aging potential.

## Introduction

Aging is a complex biological process involving the progressive decline of tissue function, resilience, and homeostatic regulation. While its hallmarks include genomic instability, mitochondrial dysfunction, inflammation, and altered energy metabolism (López-Otín et al., 2013; Franceschi and Campisi, 2014), increasing attention has turned to the role of the gut microbiota in modulating systemic aging mechanisms (Cryan et al., 2019; Bourrie et al., 2016; Farnworth, 2005). Fermented foods—particularly those containing diverse probiotic communities—have been proposed as dietary modulators of host-microbiota interactions (Marsh et al., 2013). Among them, milk kefir, a traditional fermented beverage produced by inoculating milk with complex microbial consortia (kefir grains), has long been regarded as a functional food with broad health potential (Marsh et al., 2013). First proposed by 1908 Medicine Nobel price E. Metchnikoff as a factor in human longevity (Metchnikoff, 1908), kefir is now being re-examined in the light of modern microbiome science (Vinderola et al., 2019).

Yet, despite its widespread use and probiotic richness, kefir remains underrepresented in targeted aging research. While several studies suggest beneficial effects on oxidative stress, immune function, metabolic regulation, and gut integrity (Kairey et al., 2022; Vinderola et al., 2019), the literature is scattered and lacks a unified framework aligned with contemporary models of aging.

In this Perspective, we apply AI-assisted evidence mapping to systematically classify the current scientific literature on kefir and aging according to four functional domains described by Colloca et al. (2020): changes in body composition, neurodegeneration, energy balance, and signaling networks for homeostasis. Rather than exhaustively cataloguing findings, our goal is to identify thematic imbalances and underexplored domains, offering a conceptual map to guide future research into kefir’s anti-aging potential. This strategy was specifically chosen to overcome the scarcity of direct studies explicitly linking kefir consumption with aging outcomes. By adopting a domain-based clustering framework, we were able to capture biologically relevant connections that may not be immediately apparent through traditional keyword-based searches, thus broadening the scope of evidence mapping and maximizing the translational relevance of the identified research gaps.

### State of the art by aging domains

To address the heterogeneity of biological mechanisms involved in aging, we adopted the functional classification proposed by Colloca et al., which distinguishes four interconnected domains: changes in body composition, energy balance, homeostasis signaling networks, and neurodegeneration. This taxonomy provides a conceptual framework that reflects both the physiological impact of aging and its clinical manifestations. Mapping the effects of kefir within these domains allows for a more structured interpretation of the literature, highlights neglected outcomes, and supports alignment with scientific priorities.

#### Changes in body composition

Alterations in body composition—such as reduced muscle mass, increased fat accumulation, and bone mineral density loss—are central to aging and frailty. Osteoporosis, a quintessential example, leads to fragility fractures and accounts for significant global morbidity and healthcare costs (Cruz-Jentoft et al., 2019; Burge et al., 2007). Aging exacerbates these changes via hormonal dysregulation, impaired calcium absorption, and chronic inflammation. Kefir, rich in calcium and often fortified with vitamin D, has been explored for its potential in mitigating bone loss and sarcopenia. In clinical trials, kefir has been associated with improved bone turnover markers and increases in bone mineral density (BMD), especially in postmenopausal women (Curciarello et al., 2021). In murine models, probiotics like *Lactobacillus reuter*i and *Lactobacillus kefiri*, found in kefir, significantly improved femoral BMD and suppressed inflammatory cytokines such as TNF-α and IL-6 (Collins et al., 2016; Li et al., 2016; Iraporda et al., 2014). Mechanistically, kefir may support musculoskeletal health during aging by enhancing calcium absorption, modulating parathyroid hormone (PTH) activity, and promoting osteoblast differentiation (Collins et al., 2016; Li et al., 2016). These effects are often accompanied by suppression of pro-resorptive cytokines such as TNF-α, supporting the preservation of bone mineral density under inflammatory conditions (Curciarello et al., 2021).

#### Energy balance

The balance between energy intake and expenditure becomes increasingly fragile with age, influencing both longevity and metabolic resilience. Several studies have reported that kefir improves glucose metabolism and insulin sensitivity in aging models, possibly through microbiota modulation and zonulin downregulation (Ton et al., 2020; Praznikar et al., 2020); while in another study increase Apo1 protein, exerting anti-inflammatory properties (Bellikci-Koyu et al., 2022). In cancer survivors and elderly patients, kefir improved lean mass, reduced fatigue, and decreased Lipopolysaccharide (LPS) levels (Brasiel et al., 2022). Also, modulation of adipokines and cytokines in obesity links kefir to delayed metabolic syndrome (Ouchi et al., 2011). In particular, Apo1 protein is considered an anti-inflammatory mediator that may contribute to these beneficial effects. Despite these findings, energy balance remains underrepresented in kefir and aging research. Underlying these metabolic outcomes, kefir has been shown to activate AMP-activated protein kinase (AMPK) and potentially suppress mTOR signaling (El Sayed et al., 2022). These molecular events coincide with improved insulin sensitivity and beneficial modulation of adipokines, such as adiponectin and leptin (Ton et al., 2020; Praznikar et al., 2020; Ouchi et al., 2011), contributing to the restoration of metabolic flexibility in aging models.

#### Homeostasis signaling networks

Homeostatic signaling is the most explored in the context of kefir. Homeostasis is the capability of a system to regulate its internal surroundings through maintaining a stable, relatively regular set of properties such as temperature and ph. This includes oxidative stress responses, immune function, and inflammation—all central to inflammaging (Franceschi and Campisi, 2014; Ouchi et al., 2011). Kefir and its microbes enhance antioxidant defenses (e.g., superoxide dismutase [SOD], catalase [CAT]), reduce reactive oxygen species (ROS), and modulate cytokines (Fulop et al., 2018). These effects appear across models, from cell lines to humans. Telomere attrition, mitochondrial dysfunction, and senescence are also impacted. Kefir modulates the gut–immune axis and redox balance, suggesting a systemic benefit (Ahmed et al., 2021; Rosa et al., 2020).

#### Neurodegeneration

Despite compelling hypotheses, kefir’s role in neurodegeneration is underexplored, and relies mostly on reviews. Alzheimer’s disease (AD) involves inflammation, microglial activation, and ROS, all microbiota-sensitive (Heneka et al., 2015; Sharon et al., 2016). One study reported that kefir improved the global cognitive status of patients with Alzheimer’s disease. Although the sample size was limited, the intervention resulted in cognitive improvement in 28% of the participants, but no neuroimaging or biomarker-based validation was included (Ton et al., 2020). Animal models confirm neuroprotection, showing improved memory and reduced anxiety-like behavior (Ribeiro et al., 2018). Yet, human trials are lacking, making this the most neglected kefir-aging interface. Emerging preclinical evidence suggests that kefir may exert neuroprotective effects through upregulation of brain-derived neurotrophic factor (BDNF) and modulation of microglial activation (Rosa et al., 2020; Heneka et al., 2015). These effects are likely mediated, at least in part, via the gut–brain axis, as kefir-derived probiotics influence microbial metabolites and neuroinflammatory signaling. While epigenetic mechanisms, such as telomere attrition, have been proposed in aging-related neurodegeneration, they remain unexplored in the context of kefir. This may contribute to attenuation of cognitive decline, as observed in early-phase clinical studies (Sharon et al., 2016).

### Results as evidence map

**FIGURE 1:**
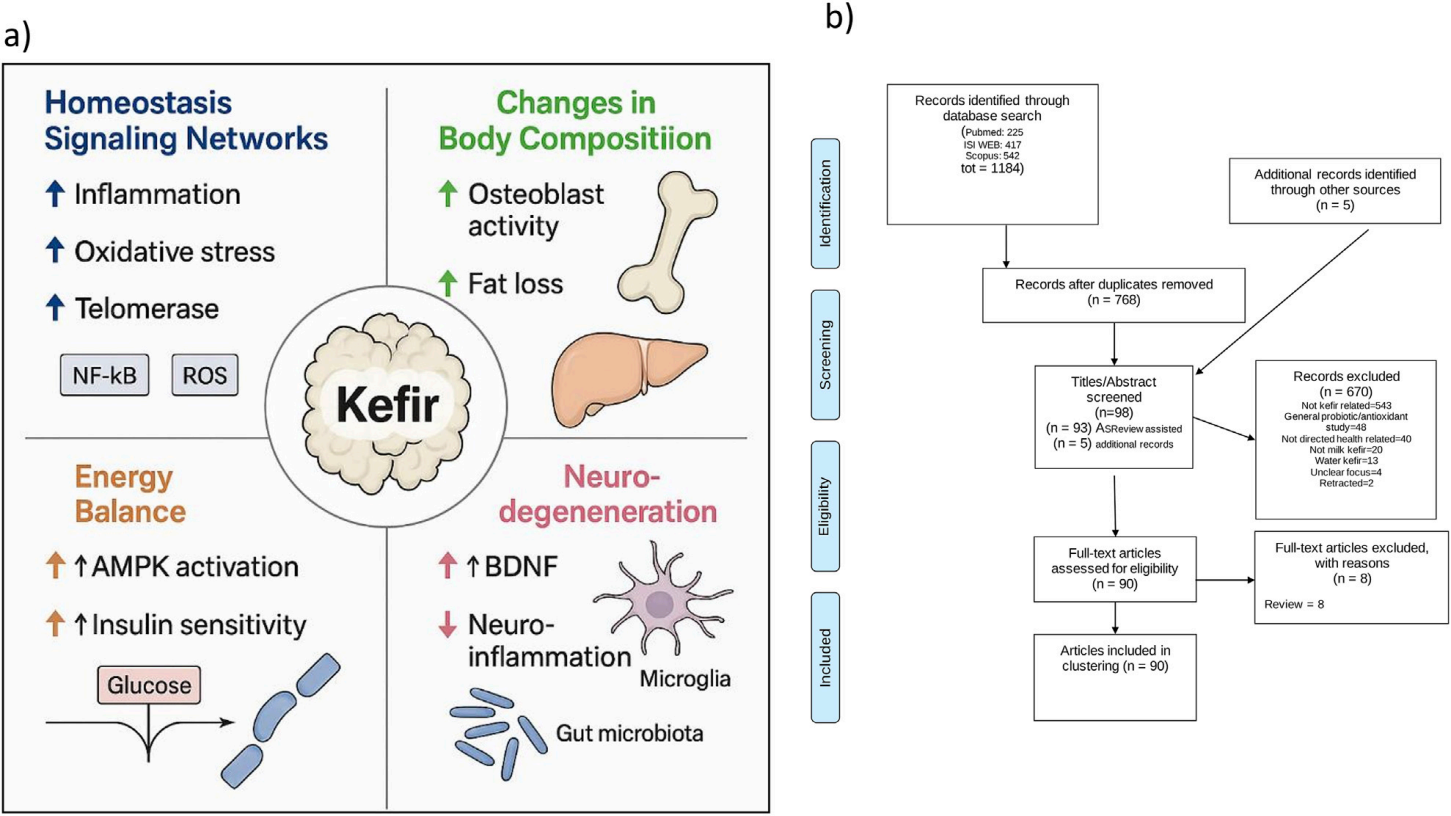
Conceptual and methodological framework of kefir-aging literature mapping. **(a)** Schematic of kefir’s effects across aging domains. Visual representation of the proposed multisystemic actions of kefir across four functional domains—Homeostasis Signaling Networks, Changes in Body Composition, Energy Balance, and Neurodegeneration—based on evidence extracted from the selected literature. Key pathways include modulation of inflammation (↓ IL-6, TNF-α; ↑ IL-10), redox balance (↑ SOD, CAT; ↓ ROS), metabolic signaling (↑ AMPK, insulin sensitivity; ↓ mTOR, zonulin), bone regulation (↑ BMD, osteoblast activity), and neuroprotection (↑ BDNF; gut–brain axis modulation; ↓ neuroinflammation). **(b)** *PRISMA flow diagram.* Summary of the AI-assisted literature screening process, including database retrieval, deduplication, inclusion via ASReview algorithm, and final full-text selection, resulting in 90 studies classified into the four aging domains.

To conceptually frame the biological relevance of kefir in aging, we first present an integrative schematic (Figure 1a) illustrating its potential multisystemic effects across the four functional domains used in this review.

The literature selection process is then summarized in the PRISMA (Page et al., 2021) flow diagram (Figure 1b), detailing database retrieval, deduplication, AI-assisted inclusion (via ASReview), and full-text evaluation steps, which resulted in 90 articles selected for thematic classification. A total of 1,184 records were retrieved from three major databases: PubMed (n = 225), ISI Web of Science (n = 417), and Scopus (n = 542). The search strings are declared in Supplementary data 1 (SD1). After deduplication, 768 unique articles remained. Five additional records were manually added. Screening was conducted using a AI-assisted algorithm “ASReview” (van de Schoot et al., 2021) (SD2), resulting in 93 records selected for inclusion, of which 90 met the criteria after full-text assessment (SD3: complete database). In addition to studies conducted in vertebrate models using traditional grain-derived kefir, this systematic review also included investigations focusing on the primary chemical constituents of kefir, as well as studies evaluating individual microbial strains commonly isolated from kefir. These strains included *Candida kefyr, Kluyveromyces marxianus subsp. marxianus, Lactobacillus acidophilus, Lactobacillus delbrueckii subsp. bulgaricus, Lactobacillus kefiranofaciens, Lacticaseibacillus paracasei, Lactiplantibacillus plantarum, Saccharomyces cerevisiae, and Saccharomyces unisporus* (Prado et al., 2015).

**FIGURE 2:**
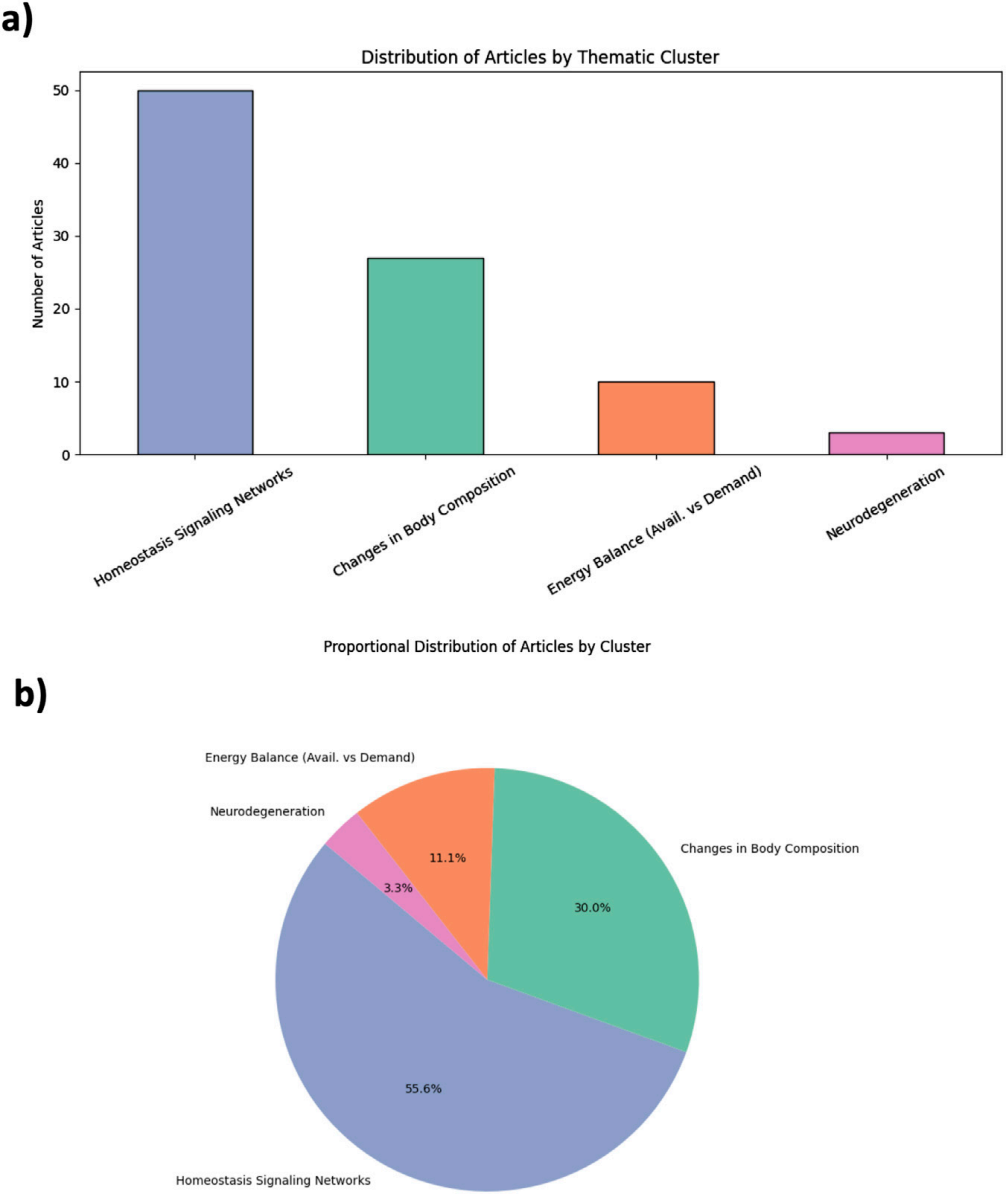
Thematic distribution of kefir and aging studies based on AI-guided evidence mapping. **(a)** Bar plot showing the number of studies mapped to each aging domain: Homeostasis Signaling Networks, Changes in Body Composition, Energy Balance, and Neurodegeneration. **(b)** Pie chart illustrating proportional representation across the four domains. The classification is based on semantic keyword clustering applied to abstracts. Each study was assigned to a single domain based on semantic proximity to predefined vocabularies (see Supplementary Table S2). This strategy prioritizes thematic clarity over mechanistic overlap.

The 90 included studies were thematically classified into four domains of aging according to the framework of Colloca et al. This classification was based on a Python-based Python-based semantic analysis pipeline that identified domain-specific keywords within abstracts and titles, as provided in Supplementary Data 4 (SD4: Python script - Jupyter Notebook version). To complement the AI-assisted screening process, the thematic mapping of the selected studies was conducted using a keyword-based semantic clustering strategy. Predefined vocabularies corresponding to the four aging domains proposed by Colloca et al. were applied to the titles and abstracts of the included articles. This rule-based semantic approach does not rely on machine learning algorithms, but provides a structured, reproducible framework to map the current literature landscape and identify thematic biases. We acknowledge that this strategy does not assess the methodological quality of the studies, nor does it replace expert-driven critical synthesis. However, it enables the identification of evidence gaps and research imbalances that merit further exploration. The distribution of articles across the four domains is illustrated in Figures 2a,b.

A predominant share of studies clustered under the domain of Homeostasis Signaling Networks (n = 50; 55.6%), with a strong focus on inflammation, oxidative stress, and immune pathways. These studies often evaluated cytokine profiles (e.g., Interleukine 6 [IL-6], tumor necrosis factor alpha [TNF-α]), redox balance, and anti-inflammatory properties of kefir or its microbial components. Changes in Body Composition accounted for 27 studies (30%), including research on sarcopenia, bone mineral density, and muscle mass preservation. Energy Balance was explored in 12 studies (11.1%), mostly through metabolic parameters such as insulin sensitivity or lipid metabolism. Only three studies (3.3%) addressed the domain of Neurodegeneration, highlighting the major underrepresentation of neurological outcomes in kefir-related aging research. This pronounced thematic asymmetry reflects a mechanistic bias, favoring inflammation-related molecular pathways while overlooking integrative physiological and cognitive domains. The complete list of included articles, along with titles, keywords, and cluster assignments, is provided as Supplementary Table S2 (ST1), while the vocabulary for classification is defined in the same supplementary file as (ST2).

## Discussion

Thematic clustering of 90 studies, spanning publications from 1993 to early 2025, revealed a sensible imbalance in the literature exploring kefir’s relationship with aging. Over half of the studies were concentrated in the domain of homeostasis signaling networks, focusing predominantly on inflammation, oxidative stress, and immune modulation. These are indeed central pathways in aging biology (López-Otín et al., 2013; Franceschi and Campisi, 2014), but their overrepresentation likely reflects a mechanistic bias in the current research paradigm. While molecular mechanisms are important, the relative under representation of domains such as energy balance (11.1%) and neurodegeneration (3.3%) indicates a structural limitation in scope. Notably, domains with direct clinical implications—such as cognitive decline or metabolic flexibility—remain underexplored, despite growing evidence of kefir’s potential to influence gut–brain signaling and metabolic regulation (Cryan et al., 2019; Bourrie et al., 2016; Farnworth, 2005). Neurodegeneration was the least represented domain. This likely reflects both the difficulty in modeling cognitive aging and the underuse of neuroimaging or validated biomarkers in kefir research. Moreover, most studies in this domain were either preclinical or observational, limiting causal inference and clinical applicability.

We propose that future studies integrate longitudinal designs, assess neurocognitive endpoints, and explore inter-domain interactions—such as those between inflammation and sarcopenia, or along the gut–brain and gut–metabolism axes—which may mediate kefir’s multisystemic effects.

Moreover, efforts should be made to standardize kefir preparations, as variations between traditional and industrial formulations may confound biological outcomes (Bourrie et al., 2023).

This Perspective does not aim to provide final answers, but rather to offer a map—constructed with AI support—that reveals where the literature is concentrated and, more importantly, where it remains silent.

To better contextualize this thematic asymmetry, we summarized the domain-specific mechanistic pathways emerging from the included studies in Table 1. This overview highlights both well-characterized molecular actions—especially in the domain of homeostasis—and the relative scarcity of mechanistic insights in domains like energy regulation and neurodegeneration.

**TABLE 1:** Mechanistic pathways of kefir across aging domains.

Domain	Key mechanisms identified in literature	Evidence level; [References]	Notable gaps/Underrepresented areas
Homeostasis Signaling Networks	↓ TNF-α, IL-6, ↑ IL-10, modulation of NF-κB and Nrf2 pathways; ↑ antioxidant enzymes (SOD, CAT), ↓ ROS; regulation of gut–immune axis; telomere maintenance; ↓ systemic inflammation	High (50/90 studies) (Curciarello et al., 2021; Albuquerque Pereira et al., 2022; Albuquerque Pereira et al., 2021; Erdogan et al., 2015; Franco et al., 2015; Coco et al., 2018; Rasipin et al., 2020; Hamida et al., 2021; Melo et al., 2014; Ali et al., 2019; de Almeida Silva et al., 2020; El et al., 2017; Topuz et al., 2018; Raras et al., 2019; Huseini et al., 2012; Rahimzadeh et al., 2019; Thoreux et al., 2015; Yasar et al., 2018; Santanna et al., 2021; Mendes et al., 2021; Ekici et al., 2022; Aslan et al., 2019; Lee et al., 2020; Pektas et al., 2021; Chuang et al., 2019; Rodrigues et al., 2020; Tung et al., 2021; Cui et al., 2024; Chen et al., 2019; Seo et al., 2021; Chen et al., 2020a; Çıtar Dazıroğlu et al., 2024; Chen et al., 2021; Aires et al., 2022; El Sayed et al., 2021; Chen et al., 2020b; Lan et al., 2024; Salah et al., 2022; Du et al., 2021; Wang et al., 2023a; Kwon et al., 2022; Chen et al., 2024; Liao et al., 2023; Noori et al., 2023; Zeng et al., 2022; Hong et al., 2010; De Montijo-Prieto et al., 2015; O'Brien et al., 2015; Malta et al., 2023; Radhouani et al., 2018)	Few studies link these changes to longitudinal or multi-tissue functional aging outcomes
Changes in Body Composition	↑ Bone mineral density (BMD); ↑ calcium and collagen metabolism; ↓ fat mass and adipocyte inflammation; modulation of osteoblast/osteoclast activity; possible PTH and estrogen signaling involvement	Moderate (27/90 studies); (Bourrie et al., 2016); (Brasiel et al., 2022; Marquina et al., 2002; Falasca et al., 2015; da Silva et al., 2015; Vieira et al., 2013; Moazen et al., 2019; Sevencan et al., 2020; Tung et al., 2018; Choi et al., 2013; Du et al., 2020; Santos et al., 2021; Smoak et al., 2020; Zeng et al., 2021; Anwar et al., 2022; Chang et al., 2020; de Vasconcelos et al., 2022; Kim et al., 2021; Gao et al., 2020; Youn et al., 2021; Senol et al., 2020; Cho et al., 2013; Lin et al., 2021; Bae et al., 2020; Bourrie et al., 2023; Gao et al., 2022; Tu et al., 2020)	Sarcopenia poorly addressed; lack of studies assessing muscle strength, mitochondrial function
Energy Balance	↑ AMPK, ↓ mTOR (suggested); improved glucose uptake and insulin sensitivity; ↑ ApoA1; ↓ zonulin (gut permeability); modulation of adipokines (e.g., adiponectin, leptin); ↓ NAFLD progression	Limited (12/90 studies) (Bellikci-Koyu et al., 2022; El Sayed et al., 2022; Bourrie et al., 2023; Alihosseini et al., 2015; Chen et al., 2022; Prašnikar et al., 2022; Pugliero et al., 2022; Ostadrahimi et al., 2015; Zubiría et al., 2017; El-Bashiti et al., 2018)	No data on caloric restriction mimetics; energy expenditure unmeasured; unclear link to mitochondrial aging
Neurodegeneration	↓ neuroinflammation (IL-6, TNF-α); improved cognitive performance in AD models; ↑ BDNF (reported once); possible gut–brain axis modulation; ↓ oxidative damage in CNS	Very Limited (3/90 studies) (Ton et al., 2020; Anwar et al., 2023; Wang et al., 2023b)	No RCTs; lacking studies on microglia activation states, synaptic function, or human neurocognitive aging

An overview of these mechanisms, categorizing them by domain, frequency of reporting in the included literature, and key knowledge gaps. This synthesis confirms that most mechanistic insights are concentrated in inflammation- and redox-related pathways (within the Homeostasis domain), whereas domains with high clinical relevance—such as neurodegeneration or metabolic resilience—remain largely underexplored. Importantly, this table also highlights the complexity and pleiotropy of kefir’s potential biological effects, suggesting that its impact may span across multiple physiological systems in parallel. Such systemic action underscores the limitations of strictly reductionist models and supports the need for integrated evidence-mapping frameworks like the one presented in this work. Every article herein cited is present in the Bibliographic References section.

Our evidence mapping of kefir and aging literature reveals a pronounced thematic concentration on homeostasis-related signaling, to the detriment of domains like neurodegeneration and energy regulation. By applying a structured clustering model based on the aging domains proposed by Colloca et al. , we highlight the need for a broader and more clinically integrative research agenda. This includes not only exploring kefir’s molecular properties, but also assessing its translational relevance across multiple aging systems.

The approach presented here demonstrates how AI and semantic clustering can support literature synthesis and strategic research planning, offering a reproducible framework to identify gaps and reorient scientific focus in emerging health domains. The herein applied integrative pipeline, indeed, combining AI-assisted screening and semantic clustering, represents a transferable strategy that could be adapted to other functional foods or emerging research domains, maximizing discovery and guiding translational innovation. Finally, we acknowledge that the AI-assisted screening process, while efficient and reproducible, may still be susceptible to latent biases inherent in the literature, including publication bias and preferential keyword usage. These limitations reinforce the importance of expert validation and transparent classification criteria in thematic mapping studies.

## Data Availability

The original contributions presented in the study are included in the article/Supplementary Material, further inquiries can be directed to the corresponding author.
