# Supplementary material for: Kefir and healthy aging: revealing thematic gaps through AI-assisted screening and semantic evidence mapping
Source: Front Aging. 2025 Oct 2;6:1628474. doi: 10.3389/fragi.2025.1628474 (PMC12528173; doi:10.3389/fragi.2025.1628474)
Supplement: Supplementary file 2 [file Table1.docx]

**Supplementary data 1**

**String searches**

ISI web:

<https://www.webofscience.com/wos/woscc/summary/a5e81fd7-b4bb-401b-8c71-351319d48f4a-01599fd874/relevance/1>

Results for ((TS=(("kefir" OR "kephis" OR "fermented milk" OR "Tibetan mushroom"))) AND TS=("senescence" OR "ageing" OR "inflammation" OR "neuroinflammation" OR "life prolongation" OR "inflammaging" OR "inflamm aging")) AND LA=(English)

*| Timespan: 1993-01-01 to 2025-01-01 (Publication Date)*

Scopus

TITLE-ABS-KEY ( "kefir" OR "kephir" OR "fermented milk" OR "Tibetan mushroom" AND "senescence" OR "ageing" OR "inflammation" OR "neuroinflammation" OR "life prolongation" OR "inflammaging" OR "inflamm aging" ) AND PUBYEAR > 1993 AND PUBYEAR < 2025 AND ( LIMIT-TO ( LANGUAGE , "English" ) )

Pubmed

("kefir" OR "kephir" OR "fermented milk" OR "Tibetan mushroom")

AND

("senescence" OR "aging" OR "ageing" OR "inflammation" OR "neuroinflammation" OR "life prolongation" OR "inflammaging" OR "inflamm aging")

AND ("1993/01/01"[Date - Publication] : "2025/12/31"[Date - Publication])

AND (english[Language])

**Supplementary data 2**

This supplementary material provides two key outputs related to the AI-assisted screening process using ASReview:


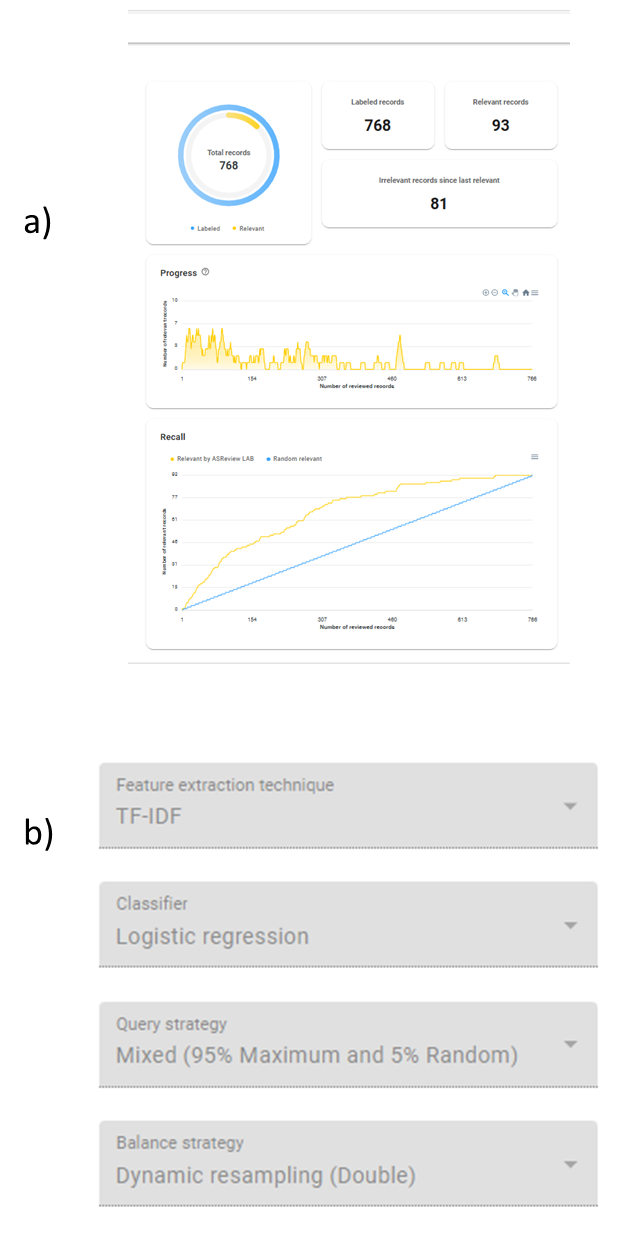


a) the final analytics generated after the completion of the screening project, including labeling performance and stopping criteria visualization;
b) a detailed summary of the machine learning model setup, including classifier selection, query strategy, balance strategy, and feature extraction method.

Manual refinement procedure: After initial semantic clustering using the predefined vocabulary described above, all included articles were manually reviewed by domain experts. When misclassifications were identified—such as studies assigned to a single domain despite clear relevance to others—new keywords were iteratively added to the domain-specific vocabularies. This allowed the system to progressively improve its classification accuracy while maintaining full transparency and reproducibility. This expert-guided review and refinement process is not encoded in machine learning models, but complements the rule-based keyword strategy outlined in this supplementary material.
