# Supplementary material for: Kefir and healthy aging: revealing thematic gaps through AI-assisted screening and semantic evidence mapping
Source: Front Aging. 2025 Oct 2;6:1628474. doi: 10.3389/fragi.2025.1628474 (PMC12528173; doi:10.3389/fragi.2025.1628474)
Supplement: Supplementary file 3 [file Table2.docx]

**Supplementary Table 1**: Summary of Included Articles and Thematic Classification

This supplementary table provides a detailed summary of the included articles, listing the record number, authors, title, assigned thematic cluster, and the matched keywords identified through AI-assisted text analysis.

| No. | Authors | Title | Thematic Cluster | Matched Keywords |
| --- | --- | --- | --- | --- |
| 1 | Marquina D.; Santos A.; Corpas I.; Muñoz J.; Zazo J.; Peinado J.M. | Dietary influence of kefir on microbial activities in the mouse bowel | Changes in Body Composition | bowel, intestinal microflora |
| 2 | Falasca K, Vecchiet J, Ucciferri C, Di Nicola M, D'Angelo C, Reale M. | Effect of Probiotic Supplement on Cytokine Levels in HIV-Infected Individuals: A Preliminary Study | Changes in Body Composition | fat |
| 3 | da Silva, KN; Fávero, AG; Ribeiro, W; Ferreira, CM; Sartorelli, P; Cardili, L; Bogsan, CS; Pereira, JNB; Sinigaglia, RD; Malinverni, ACD; Paiotti, APR; Miszputen, SJ; Ambrogini, O Jr | Effects of kefir fermented milk beverage on sodium dextran sulfate (DSS)-induced colitis in rats | Changes in Body Composition | gut, fat, bowel |
| 4 | Vieira LV, de Sousa LM, Maia TAC, Gusmão JNFM, Goes P, Pereira KMA, Miyajima F, Gondim DV. | Milk Kefir therapy reduces inflammation and alveolar bone loss on periodontitis in rats | Changes in Body Composition | fat, osteoporosis, bone, collagen |
| 5 | Moazen M, Mazloom Z, Tanideh N, Dabbaghmanesh MH, Rahmdel S, Azarpira N, Fararouei M. | Osteoprotective effects of kefir fortified with omega-3 and vitamin C in ovariectomized rats | Changes in Body Composition | fat, osteoporosis, bone, collagen |
| 6 | Sevencan, NO; Isler, M; Kapucuoglu, FN; Senol, A; Kayhan, B; Kiztanir, S; Kockar, MC | Dose-dependent effects of kefir on colitis induced by trinitrobenzene sulfonic acid in rats | Changes in Body Composition | gut, bowel |
| 7 | Tung YT, Chen HL, Wu HS, Ho MH, Chong KY, Chen CM. | Kefir Peptides Prevent Hyperlipidemia and Obesity in High-Fat-Diet-Induced Obese Rats via Lipid Metabolism Modulation | Changes in Body Composition | fat, adipose, body weight |
| 8 | Bourrie, BCT; Cotter, PD; Willing, BP | Traditional kefir reduces weight gain and improves plasma and liver lipid profiles more successfully than a commercial equivalent in a mouse model of obesity | Changes in Body Composition | gut, fat |
| 9 | Choi, JW; Kang, HW; Lim, WC; Kim, MK; Lee, IY; Cho, HY | Kefir prevented excess fat accumulation in diet-induced obese mice | Changes in Body Composition | fat, adipose, body weight |
| 10 | Du, GA; Guo, Q; Yan, XH; Chen, H; Yuan, YH; Yue, TL | Potential protective mechanism of Tibetan kefir underlying gut-derived liver injury induced by ochratoxin A | Changes in Body Composition | gut, fat |
| 11 | Santos F.R.D.; Ribeiro G.H.M.; Monteiro-Junior R.S.; Barcala-Jorge A.S.; Guimarães A.L.S.; de Paula A.M.B.; Santos S.H.S. | Supplementation with kefir improves metabolism and liver inflammation in malnourished mice | Changes in Body Composition | body composition, adipose, body weight, adiposity |
| 12 | Smoak P, Harman N, Flores V, Kisiolek J, Pullen NA, Lisano J, Hayward R, Stewart LK. | Kefir Is a Viable Exercise Recovery Beverage for Cancer Survivors Enrolled in a Structured Exercise Program | Changes in Body Composition | gut, fat |
| 13 | Zeng X, Jia H, Zhang X, Wang X, Wang Z, Gao Z, Yuan Y, Yue T. | Supplementation of kefir ameliorates azoxymethane/dextran sulfate sodium induced colorectal cancer by modulating the gut microbiota | Changes in Body Composition | gut, fat |
| 14 | Anwar, MM; Boseila, AA; Mabrouk, AA; Abdelkhalek, AA; Amin, A | Impact of Lyophilized Milk Kefir-Based Self-Nanoemulsifying System on Cognitive Enhancement via the Microbiota-Gut-Brain Axis | Changes in Body Composition | gut, body weight, bowel |
| 15 | Chang GR, Cheng WY, Fan HC, Chen HL, Lan YW, Chen MS, Yen CC, Chen CM. | Kefir peptides attenuate atherosclerotic vascular calcification and osteoporosis in atherogenic diet-fed ApoE (-/-) knockout mice | Changes in Body Composition | muscle, fat, osteoporosis, bone, collagen |
| 16 | de Vasconcelos RF, Costa V, Araujo B, Maia TAC, Dias R, Vasconcelos L, Silveira H, Carneiro B, Thiers D, Costa FWG, Kurita L, Ayala A, Leitão R, Pereira KMA, Gondim DV, Goes P. | Milk kefir therapy improves the skeletal response to resistance exercise in rats submitted to glucocorticoid-induced osteoporosis | Changes in Body Composition | osteoporosis, bone, collagen |
| 17 | Kim E, Lee HG, Han S, Seo KH, Kim H. | Effect of Surface Layer Proteins Derived from Paraprobiotic Kefir Lactic Acid Bacteria on Inflammation and High-Fat Diet-Induced Obesity | Changes in Body Composition | fat, adipose, body weight |
| 18 | Guiomar de Almeida Brasiel P, Cristina Potente Dutra Luquetti S, Dutra Medeiros J, Otavio do Amaral Corrêa J, Barbosa Ferreira Machado A, Paula Boroni Moreira A, Novaes Rocha V, Teodoro de Souza C, do Carmo Gouveia Peluzio M. | Kefir modulates gut microbiota and reduces DMH-associated colorectal cancer via regulation of intestinal inflammation in adulthood offsprings programmed by neonatal overfeeding | Changes in Body Composition | gut, adipose, adiposity |
| 19 | Gao X, Wang F, Zhao P, Zhang R, Zeng Q. | Effect of heat-killed Streptococcus thermophilus on type 2 diabetes rats | Changes in Body Composition | gut, fat |
| 20 | Youn, HY; Kim, HJ; Kim, H; Seo, KH | A comparative evaluation of the kefir yeast Kluyveromyces marxianus A4 and sulfasalazine in ulcerative colitis: anti-inflammatory impact and gut microbiota modulation | Changes in Body Composition | gut, fat, body weight |
| 21 | Senol A, Isler M, Sutcu R, Akin M, Cakir E, Ceyhan BM, Kockar MC. | Kefir treatment ameliorates dextran sulfate sodium-induced colitis in rats | Changes in Body Composition | fat, bmi |
| 22 | Cho J.H.; Zhang Z.F.; Kim I.H. | Effects of single or combined dietary supplementation of ?-glucan and kefir on growth performance, blood characteristics and meat quality in broilers | Changes in Body Composition | body weight |
| 23 | Lin YC, Chen YT, Li KY, Chen MJ. | Investigating the Mechanistic Differences of Obesity-Inducing Lactobacillus kefiranofaciens M1 and Anti-obesity Lactobacillus mali APS1 by Microbolomics and Metabolomics | Changes in Body Composition | gut, fat, body weight |
| 24 | Bae D.; Kim D.-H.; Chon J.-W.; Song K.-Y.; Seo K.-H. | Synergistic effects of the early administration of Lactobacillus kefiranofaciens DN1 and Kluyveromyces marxianus KU140723-05 on the inhibition of Salmonella Enteritidis colonization in young chickens | Changes in Body Composition | gut |
| 25 | Benjamin C.T. Bourrie, Paul D. Cotter, Benjamin P. Willing | Traditional kefir reduces weight gain and improves plasma and liver  lipid profiles more successfully than a commercial equivalent in a mouse  model of obesity | Changes in Body Composition | gut, fat |
| 26 | Gao J, Ding G, Li Q, Gong L, Huang J, Sang Y. | Tibet kefir milk decreases fat deposition by regulating the gut microbiota and gene expression of Lpl and Angptl4 in high fat diet-fed rats | Changes in Body Composition | fat, adipose |
| 27 | Tu MY, Chen HL, Tung YT, Kao CC, Hu FC, Chen CM. | Short-Term Effects of Kefir-Fermented Milk Consumption on Bone Mineral Density and Bone Metabolism in a Randomized Clinical Trial of Osteoporotic Patients | Changes in Body Composition | osteoporosis, bone, collagen |
| 28 | Bourrie BCT, Forgie AJ, Makarowski A, Cotter PD, Richard C, Willing BP. | Consumption of kefir made with traditional microorganisms resulted in greater improvements in LDL cholesterol and plasma markers of inflammation in males when compared to a commercial kefir: a randomized pilot study | Energy Balance (Avail. vs Demand) | metabolic, glucose, insulin |
| 29 | Alihosseini, N; Moahboob, SA; Farrin, N; Mobasseri, M; Taghizadeh, A; Ostadrahimi, AR | EFFECT OF PROBIOTIC FERMENTED MILK (KEFIR) ON SERUM LEVEL OF INSULIN AND HOMOCYSTEINE IN TYPE 2 DIABETES PATIENTS | Energy Balance (Avail. vs Demand) | insulin |
| 30 | Bellikci-Koyu E.; Sarer-Yurekli B.P.; Karagozlu C.; Aydin-Kose F.; Ozgen A.G.; Buyuktuncer Z. | Probiotic kefir consumption improves serum apolipoprotein A1 levels in metabolic syndrome patients: a randomized controlled clinical trial | Energy Balance (Avail. vs Demand) | metabolic |
| 31 | El Sayed NS, Kandil EA, Ghoneum MH. | Enhancement of Insulin/PI3K/Akt Signaling Pathway and Modulation of Gut Microbiome by Probiotics Fermentation Technology, a Kefir Grain Product, in Sporadic Alzheimer's Disease Model in Mice | Energy Balance (Avail. vs Demand) | metabolic, insulin, mTOR |
| 32 | Chen HL, Tsai TC, Tsai YC, Liao JW, Yen CC, Chen CM. | Kefir peptides prevent high-fructose corn syrup-induced non-alcoholic fatty liver disease in a murine model by modulation of inflammation and the JAK2 signaling pathway | Energy Balance (Avail. vs Demand) | metabolic, insulin, energy intake, ampk |
| 33 | Pranikar ZJ, Kenig S, Vardjan T, Bizjak M?, Petelin A. | Effects of kefir or milk supplementation on zonulin in overweight subjects | Energy Balance (Avail. vs Demand) | metabolic, glucose |
| 34 | Pugliero, S; Lima, DY; Rodrigues, AM; Bogsan, CSB; Rogero, MM; Punaro, GR; Higa, EMS | Kefir reduces nitrosative stress and upregulates Nrf2 in the kidney of diabetic rats | Energy Balance (Avail. vs Demand) | metabolic, glucose |
| 35 | Ostadrahimi, A; Taghizadeh, A; Mobasseri, M; Farrin, N; Payahoo, L; Gheshlaghi, ZB; Vahedjabbari, M | Effect of Probiotic Fermented Milk (Kefir) on Glycemic Control and Lipid Profile In Type 2 Diabetic Patients: A Randomized Double-Blind Placebo-Controlled Clinical Trial | Energy Balance (Avail. vs Demand) | glucose, energy intake |
| 36 | Zubiría MG, Gambaro SE, Rey MA, Carasi P, Serradell MLÁ, Giovambattista A. | Deleterious Metabolic Effects of High Fructose Intake: The Preventive Effect of Lactobacillus kefiri Administration | Energy Balance (Avail. vs Demand) | metabolic, glucose, insulin |
| 37 | Tarek A. El-Bashiti1, Baker M. Zabut, Fedaa F. Abu Safia | Effect of Probiotic Fermented Milk (Kefir) on Some Blood Biochemical  Parameters Among Newly Diagnosed Type 2 Diabetic Adult Males in Gaza  Governorate | Energy Balance (Avail. vs Demand) | glucose |
| 38 | Albuquerque Pereira MF, Morais de Ávila LG, Dos Santos Cruz BC, Almeida LF, Macedo Simões J, Campos Silva B, Pereira Aguilar A, de Oliveira LL, Vilela Gonçalves R, Ribon AOB, Mendes TAO, Gouveia Peluzio MDC. | Daily intake of household-produced milk kefir on Salmonella Typhimurium infection in C57BL/6 mice: mortality, microbiota modulation, and immunological implications | Homeostasis Signaling Networks | cytokine, inflammation, immune |
| 39 | Albuquerque Pereira MF, Morais de Ávila LG, Dos Santos Cruz BC, Campos Silva B, Licursi de Oliveira L, Vilela Gonçalves R, de Oliveira Barros Ribon A, de Oliveira Mendes TA, Gouveia Peluzio MDC. | The role of IL-10 in regulating inflammation and gut microbiome in mice consuming milk kefir and orally challenged with S. Typhimurium | Homeostasis Signaling Networks | cytokine, inflammation, ROS |
| 40 | Erdogan FS, Ozarslan S, Guzel-Seydim ZB, Kök Ta? T. | The effect of kefir produced from natural kefir grains on the intestinal microbial populations and antioxidant capacities of Balb/c mice | Homeostasis Signaling Networks | inflammation, antioxidant |
| 41 | Franco MC, Golowczyc MA, De Antoni GL, Pérez PF, Humen M, Serradell MLA. | Administration of kefir-fermented milk protects mice against Giardia intestinalis infection | Homeostasis Signaling Networks | cytokine, inflammation, immune |
| 42 | Coco, LZ; Aires, R; Carvalho, GR; Belisário, ED; Yap, MKK; Amorim, FG; Conde-Aranda, J; Nogueira, BV; Vasquez, EC; Pereira, TDC; Campagnaro, BP | Unravelling the Gastroprotective Potential of Kefir: Exploring Antioxidant Effects in Preventing Gastric Ulcers | Homeostasis Signaling Networks | antioxidant, anti-inflammatory, ROS |
| 43 | Rasipin; Dharmana E.; Hadisaputro S.; Suhartono | The effects of kefir on the inflamatory status and thyroid function (Experimental study on wistar rats after exposed to Chlorpyrifos) | Homeostasis Signaling Networks | ROS |
| 44 | Hamida RS, Shami A, Ali MA, Almohawes ZN, Mohammed AE, Bin-Meferij MM. | Kefir: A protective dietary supplementation against viral infection | Homeostasis Signaling Networks | cytokine, anti-inflammatory, il-6, immune |
| 45 | Melo, AFD; Mendonça, MCP; Rosa-Castro, RD | The protective effects of fermented kefir milk on azoxymethane-induced aberrant crypt formation in mice colon | Homeostasis Signaling Networks | Anti-proliferative, anti-inflammatory |
| 46 | Ali OSM, Amin NE, Abdel Fattah SM, Abd El-Rahman O. | Ameliorative effect of kefir against ?-irradiation induced liver injury in male rats: impact on oxidative stress and inflammation | Homeostasis Signaling Networks | inflammation, oxidative stress, antioxidant, anti-inflammatory |
| 47 | de Almeida Silva M, Mowry FE, Peaden SC, Andrade TU, Biancardi VC. | Kefir ameliorates hypertension via gut-brain mechanisms in spontaneously hypertensive rats | Homeostasis Signaling Networks | inflammation, il-6, ROS |
| 48 | El Golli-Bennour E, Timoumi R, Annaibi E, Mokni M, Omezzine A, Bacha H, Abid-Essefi S. | Protective effects of kefir against deltamethrin-induced hepatotoxicity in rats | Homeostasis Signaling Networks | inflammation, antioxidant, immune |
| 49 | Topuz E, Derin D, Can G, Kürklü E, Cinar S, Aykan F, Cevikba? A, Di?çi R, Durna Z, Sakar B, Saglam S, Tanyeri H, Deniz G, Gürer U, Ta? F, Guney N, Aydiner A. | Effect of oral administration of kefir on serum proinflammatory cytokines on 5-FU induced oral mucositis in patients with colorectal cancer | Homeostasis Signaling Networks | cytokine, immune, ROS |
| 50 | Raras T.Y.M.; Hidayati N.; Wardhani S.O. | High doses of kefir accelerate lung-injury progression in bleomycin-induced pneumonitis in rats | Homeostasis Signaling Networks | cytokine, inflammation, il-6, ROS |
| 51 | Huseini HF, Rahimzadeh G, Fazeli MR, Mehrazma M, Salehi M. | Evaluation of wound healing activities of kefir products | Homeostasis Signaling Networks | inflammation, anti-inflammatory |
| 52 | Rahimzadeh, G; Fazeli, MR; Mozafari, NA; Mesbahi, M | EVALUATION OF ANTI-MICROBIAL ACTIVITY AND WOUND HEALING OF KEFIR | Homeostasis Signaling Networks | inflammation |
| 53 | Thoreux K, Schmucker DL. | Kefir milk enhances intestinal immunity in young but not old rats | Homeostasis Signaling Networks | immune |
| 54 | Yasar M, Taskin AK, Kaya B, Aydin M, Ozaydin I, Iskender A, Erdem H, Ankatali H, Kandis H. | The early anti-inflammatory effect of Kefir in experimental corrosive esophagitis | Homeostasis Signaling Networks | inflammation, anti-inflammatory, ROS |
| 55 | Santanna, AF; Filete, PF; Lima, EM; Porto, ML; Meyrelles, SS; Vasquez, EC; Endringer, DC; Lenz, D; Abdalla, DSP; Pereira, TMC; Andrade, TU | Chronic administration of the soluble, nonbacterial fraction of kefir attenuates lipid deposition in LDLr-/- mice | Homeostasis Signaling Networks | cytokine, ROS |
| 56 | Mendes E, Casaro MB, Fukumori C, Ribeiro WR, Dos Santos AL, Sartorelli P, Lazarini M, Bogsan CSB, Oliveira MA, Ferreira CM. | Preventive oral kefir supplementation protects mice from ovariectomy-induced exacerbated allergic airway inflammation | Homeostasis Signaling Networks | inflammation, antioxidant, ROS |
| 57 | Ekici Ö, Aslan E, Alada? T, Güzel H, Korkmaz ÖA, Bostanc? A, Sadi G, Pekta? MB. | Masseter muscle and gingival tissue inflammatory response following treatment with high-fructose corn syrup in rats: Anti-inflammatory and antioxidant effects of kefir | Homeostasis Signaling Networks | inflammation, antioxidant, ROS |
| 58 | Aslan E.; Sadi G.; Guzel H.; Karaca C.; Korkmaz O.A.; Pektas M.K.; Celegen M.; Aladag T.; Oncu S.; Pektas M.B. | Kefir Prevents Adipose Tissue Growth Through the Induction of Apoptotic Elements in High-Fructose Corn Syrup-Fed Rats | Homeostasis Signaling Networks | signaling, inflammation, ROS |
| 59 | Lee MY, Ahn KS, Kwon OK, Kim MJ, Kim MK, Lee IY, Oh SR, Lee HK. | Anti-inflammatory and anti-allergic effects of kefir in a mouse asthma model | Homeostasis Signaling Networks | cytokine, inflammation, anti-inflammatory |
| 60 | Pektas, MB; Aslan, E; Güzel, H; Korkmaz, ÖA; Çelegen, K; Pektas, A; Bostanci, A; Sadi, G | Kefir protects the liver against high fructose corn syrup induced phosphodiesterase hyperactivity | Homeostasis Signaling Networks | signaling, inflammation |
| 61 | Chuang KC, Lai YW, Ko CH, Yen CC, Chen HL, Lan YW, Chen CF, Chen W, Chen CM. | Therapeutic effects of kefir peptides on adjuvant-induced arthritis in rats through anti-inflammation and downregulation of matrix metalloproteinases | Homeostasis Signaling Networks | cytokine, inflammation, anti-inflammatory, immune, ROS |
| 62 | Rodrigues K.L.; Carvalho J.C.T.; Schneedorf J.M. | Anti-inflammatory properties of kefir and its polysaccharide extract | Homeostasis Signaling Networks | anti-inflammatory |
| 63 | Tung, MC; Lan, YW; Li, HH; Chen, HL; Chen, SY; Chen, YH; Lin, CC; Tu, MY; Chen, CM | Kefir peptides alleviate high-fat diet-induced atherosclerosis by attenuating macrophage accumulation and oxidative stress in ApoE knockout mice | Homeostasis Signaling Networks | oxidative stress, immune, ROS |
| 64 | Cui, YY; Jing, C; Yue, Y; Ning, MG; Chen, H; Yuan, YH; Yue, TL | Kefir Ameliorates Alcohol-Induced Liver Injury Through Modulating Gut Microbiota and Fecal Bile Acid Profile in Mice | Homeostasis Signaling Networks | homeostasis, inflammation, oxidative stress, antioxidant |
| 65 | Chen, HL; Hung, KF; Yen, CC; Laio, CH; Wang, JL; Lan, YW; Chong, KY; Fan, HC; Chen, CM | Kefir peptides alleviate particulate matter &lt;4 ?m (PM4.0)-induced pulmonary inflammation by inhibiting the NF-kB pathway using luciferase transgenic mice | Homeostasis Signaling Networks | signaling, cytokine, inflammation, antioxidant, anti-inflammatory, il-6, immune, ROS |
| 66 | Seo MK, Park EJ, Ko SY, Choi EW, Kim S. | Therapeutic effects of kefir grain Lactobacillus-derived extracellular vesicles in mice with 2,4,6-trinitrobenzene sulfonic acid-induced inflammatory bowel disease | Homeostasis Signaling Networks | signaling, cytokine, inflammation, ROS |
| 67 | Chen MY, Wu HT, Chen FF, Wang YT, Chou DL, Wang GH, Chen YP. | Characterization of Tibetan kefir grain-fermented milk whey and its suppression of melanin synthesis | Homeostasis Signaling Networks | inflammation, antioxidant, anti-inflammatory, ROS |
| 68 | Ç?tar Daz?ro?lu ME, Acar Tek N, Cevher Akdulum MF, Y?lmaz C, Yal?nay AM. | Effects of kefir consumption on gut microbiota and health outcomes in women with polycystic ovary syndrome | Homeostasis Signaling Networks | cytokine, inflammation, il-6 |
| 69 | Chen CF, Li HP, Chao YH, Tu MY, Yen CC, Lan YW, Yang SH, Chong KY, Lin CC, Chen CM. | Suppression of Dendritic Cell Maturation by Kefir Peptides Alleviates Collagen-Induced Arthritis in Mice | Homeostasis Signaling Networks | signaling, cytokine, inflammation, immune, ROS |
| 70 | Aires R, Gobbi Amorim F, Côco LZ, da Conceição AP, Zanardo TÉC, Taufner GH, Nogueira BV, Vasquez EC, Melo Costa Pereira T, Campagnaro BP, Dos Santos Meyrelles S. | Use of kefir peptide (Kef-1) as an emerging approach for the treatment of oxidative stress and inflammation in 2K1C mice | Homeostasis Signaling Networks | antioxidant, anti-inflammatory, ROS |
| 71 | El Sayed NS, Kandil EA, Ghoneum MH. | Probiotics Fermentation Technology, a Novel Kefir Product, Ameliorates Cognitive Impairment in Streptozotocin-Induced Sporadic Alzheimer's Disease in Mice | Homeostasis Signaling Networks | cytokine, inflammation, oxidative stress |
| 72 | Chen YH, Chen HL, Fan HC, Tung YT, Kuo CW, Tu MY, Chen CM. | Anti-Inflammatory, Antioxidant, and Antifibrotic Effects of Kefir Peptides on Salt-Induced Renal Vascular Damage and Dysfunction in Aged Stroke-Prone Spontaneously Hypertensive Rats | Homeostasis Signaling Networks | cytokine, inflammation, oxidative stress, anti-inflammatory, ROS |
| 73 | Lan YW, Chen YC, Yen CC, Chen HL, Tung MC, Fan HC, Chen CM. | Kefir peptides mitigate bleomycin-induced pulmonary fibrosis in mice through modulating oxidative stress, inflammation and gut microbiota | Homeostasis Signaling Networks | inflammation, oxidative stress, anti-inflammatory, ROS |
| 74 | Salah N, Eissa S, Mansour A, El Magd NMA, Hasanin AH, El Mahdy MM, Hassan MK, Matboli M. | Evaluation of the role of kefir in management of non-alcoholic steatohepatitis rat model via modulation of NASH linked mRNA-miRNA panel | Homeostasis Signaling Networks | signaling, inflammation, ROS |
| 75 | Du G, Chang S, Guo Q, Yan X, Chen H, Shi K, Yuan Y, Yue T. | Protective effects of Tibetan kefir in mice with ochratoxin A-induced cecal injury | Homeostasis Signaling Networks | signaling, inflammation, oxidative stress, antioxidant, anti-inflammatory |
| 76 | Wang SY, Huang RF, Ng KS, Chen YP, Shiu JS, Chen MJ. | Co-Culture Strategy of Lactobacillus kefiranofaciens HL1 for Developing Functional Fermented Milk | Homeostasis Signaling Networks | antioxidant |
| 77 | Kwon OK, Ahn KS, Lee MY, Kim SY, Park BY, Kim MK, Lee IY, Oh SR, Lee HK. | Inhibitory effect of kefiran on ovalbumin-induced lung inflammation in a murine model of asthma | Homeostasis Signaling Networks | inflammation |
| 78 | Chen W, Wang J, Du L, Chen J, Zheng Q, Li P, Du B, Fang X, Liao Z. | Kefir microbiota and metabolites stimulate intestinal mucosal immunity and its early development | Homeostasis Signaling Networks | homeostasis, inflammation, immune |
| 79 | Liao CH, Yen CC, Chen HL, Liu YH, Chen YH, Lan YW, Chen KR, Chen W, Chen CM. | Novel Kefir Exopolysaccharides (KEPS) Mitigate Lipopolysaccharide (LPS)-Induced Systemic Inflammation in Luciferase Transgenic Mice through Inhibition of the NF-kB Pathway | Homeostasis Signaling Networks | signaling, antioxidant, anti-inflammatory, il-6, immune |
| 80 | Noori M, Shateri Z, Babajafari S, Eskandari MH, Parastouei K, Ghasemi M, Afshari H, Samadi M. | The effect of probiotic-fortified kefir on depression, appetite, oxidative stress, and inflammatory parameters in Iranian overweight and obese elderly: a randomized, double-blind, placebo-controlled clinical trial | Homeostasis Signaling Networks | inflammation, oxidative stress, antioxidant |
| 81 | Curciarello, R; Canziani, KE; Salto, I; Romero, EB; Rocca, A; Doldan, I; Peton, E; Brayer, S; Sambuelli, AM; Goncalves, S; Tirado, P; Correa, GJ; Yantorno, M; Garbi, L; Docena, GH; Serradell, MD; Muglia, CI | Probiotic Lactobacilli Isolated from Kefir Promote Down-Regulation of Inflammatory Lamina Propria T Cells from Patients with Active IBD | Homeostasis Signaling Networks | cytokine, anti-inflammatory, il-6, immune |
| 82 | Zeng X, Li J, Wang X, Liu L, Shen S, Li N, Wang Z, Yuan Y, Yue T. | Regulation of Gut Microbiota and Microbial Metabolome of Kefir Supernatant against Fusobacterium nucleatum and DSS-Coinduced Colitis | Homeostasis Signaling Networks | cytokine, inflammation, oxidative stress, anti-inflammatory, il-6 |
| 83 | Hong WS, Chen YP, Dai TY, Huang IN, Chen MJ. | Effect of heat-inactivated kefir-isolated Lactobacillus kefiranofaciens M1 on preventing an allergic airway response in mice | Homeostasis Signaling Networks | cytokine, inflammation |
| 84 | De Montijo-Prieto S.; Moreno E.; Bergillos-Meca T.; Lasserrot A.; Ruiz-López M.-D.; Ruiz-Bravo A.; Jiménez-Valera M. | A Lactobacillus plantarum strain isolated from kefir protects against intestinal infection with Yersinia enterocolitica O9 and modulates immunity in mice | Homeostasis Signaling Networks | cytokine |
| 85 | O'Brien KV, Stewart LK, Forney LA, Aryana KJ, Prinyawiwatkul W, Boeneke CA. | The effects of postexercise consumption of a kefir beverage on performance and recovery during intensive endurance training | Homeostasis Signaling Networks | inflammation, oxidative stress, anti-inflammatory |
| 86 | Serena Mares Malta, Letícia Leandro Batista, Heitor Cappato Guerra Silva, Rodrigo Rodrigues Franco, Matheus Henrique Silva, Tamiris Sabrina Rodrigues, Lucas Ian Veloso Correia, Mário Machado Martins, Gabriela Venturini, Foued Salmen Espindola, Murilo Vieira da Silva & Carlos Ueira-Vieira | Identification of bioactive peptides from a Brazilian kefir sample, and their anti-Alzheimer potential in Drosophila melanogaster | Homeostasis Signaling Networks | oxidative stress, antioxidant, ROS |
| 87 | Hajer Radhouani, Cristiana Gonçalves, F. Raquel Maia, Joaquim M. Oliveira & Rui L. Reis | Biological performance of a promising Kefiran-biopolymer with potential in regenerative medicine applications: a comparative study with hyaluronic acid. | Homeostasis Signaling Networks | antioxidant, anti-inflammatory |
| 88 | Anwar, MM; Ali, OSM; Ahmed, RL; Badawi, AM; Eltablawy, NA | The effect of using kefir grains and mesenchymal stem cells in LPS-induced Alzheimer's disease neuroinflammatory model | Neurodegeneration | brain, cognitive, bdnf |
| 89 | Ton AMM, Campagnaro BP, Alves GA, Aires R, Côco LZ, Arpini CM, Guerra E Oliveira T, Campos-Toimil M, Meyrelles SS, Pereira TMC, Vasquez EC. | Oxidative Stress and Dementia in Alzheimer's Patients: Effects of Synbiotic Supplementation | Neurodegeneration | cognitive, memory, dementia, neuropathological, language |
| 90 | Yuanwang Wang, Dehua Wang, Houjiao Lv, Qinchen Dong, Jiajia Li, Weitao Geng, Jinju Wang, Fufeng Liu, Longgang Jia, Yanping Wang | Modulation of the gut microbiota and glycometabolism by a probiotic to alleviate amyloid accumulation and cognitive impairments in AD rats | Neurodegeneration | brain, neuron, cognitive |

**Supplementary Table 2: Semantic Vocabulary Associated with Aging Domains**

This vocabulary was developed based on biological relevance to aging, as defined by Colloca et al. [10], and includes terms associated with body composition, energy balance, homeostasis signaling, and neurodegeneration. This structured vocabulary supports reproducibility and transparency in the thematic mapping process.

| Aging Domain | Associated Keywords |
| --- | --- |
| Changes in Body Composition | muscle, gut, fat, lean mass, body composition, sarcopenia, adipose, body weight, adiposity, bmi, bowel, intestinal microflora, osteoporosis, bone, collagen |
| Energy Balance (Avail. vs Demand) | metabolism, metabolic, caloric restriction, glucose, insulin, energy balance, energy intake, ampk, mTOR, mitochondria |
| Homeostasis Signaling Networks | homeostasis, signaling, cytokine, sirt, inflammation, oxidative stress, antioxidant, Anti-proliferative, anti-inflammatory, anti-mutagenic, nf-kb, il-6, immune, stress response, ROS |
| Neurodegeneration | neurodegeneration, brain, neuron, cognitive, memory, bdnf, hippocampus, neuroprotective, dementia, neuropathological, language |

Note on refinement:

- After initial application of this vocabulary to the included studies, a manual expert review was conducted. Articles that appeared misclassified were reassessed, and when necessary, additional keywords were incorporated into the domain-specific vocabularies. This iterative refinement ensured better alignment with the biological content of the studies and increased the robustness of the semantic classification.
- Some aging terms such as “senescence” and “inflammaging” were included in the initial search but excluded from domain-specific semantic dictionaries due to their cross-domain nature.
