# Supplementary material for: Kefir and healthy aging: revealing thematic gaps through AI-assisted screening and semantic evidence mapping
Source: Front Aging. 2025 Oct 2;6:1628474. doi: 10.3389/fragi.2025.1628474 (PMC12528173; doi:10.3389/fragi.2025.1628474)
Supplement: Supplementary file 4 [file Table4.docx]

**Supplementary data 4- python script**

import pandas as pd

import matplotlib.pyplot as plt

import os

# === 1. Load ASReview Excel file

file_path = "last_clusterization_28_4_25.xlsx" # Change this if needed

df = pd.read_excel(file_path)

df_included = df[df["included"] == 1].copy()

df_included.reset_index(drop=True, inplace=True)

# === 2. Colloca et al. thematic domains and keywords

clusters_keywords = {

"Changes in Body Composition": [

"muscle","gut" ,"fat", "lean mass", "body composition", "sarcopenia", "adipose", "body weight", "adiposity", "bmi", "bowel", "intestinal microflora", "osteoporosis", "bone", "collagen"

],

"Energy Balance (Avail. vs Demand)": [

"metabolism","metabolic", "caloric restriction", "glucose", "insulin", "energy balance", "energy intake", "ampk", "mTOR", "mitochondria"

],

"Homeostasis Signaling Networks": [

"homeostasis", "signaling", "cytokine", "sirt", "inflammation", "oxidative stress", "antioxidant","Anti-proliferative", "anti-inflammatory","anti-mutagenic"

"nf-kb", "il-6", "immune", "stress response", "ROS"

],

"Neurodegeneration": [

"neurodegeneration", "brain", "neuron", "cognitive", "memory", "bdnf", "hippocampus", "neuroprotective", "dementia", "neuropathological", "language"

]

}

# === 3. Assign cluster and matched keywords

def assign_cluster_and_keywords(text):

if pd.isna(text):

return "Unclassifiable", []

text = text.lower()

matched_keywords = {

cluster: [kw for kw in keywords if kw.lower() in text]

for cluster, keywords in clusters_keywords.items()

}

best_cluster = max(matched_keywords, key=lambda c: len(matched_keywords[c]))

best_keywords = matched_keywords[best_cluster]

if len(best_keywords) == 0:

return "Unclassifiable", []

return best_cluster, best_keywords

df_included["abstract_fallback"] = df_included["abstract"].fillna(df_included["title"])

df_included[["Thematic Cluster", "Matched Keywords"]] = df_included["abstract_fallback"].apply(

lambda x: pd.Series(assign_cluster_and_keywords(x))

)

# === 4. Colors per cluster (consistent between charts)

cluster_colors = {

"Changes in Body Composition": "#66c2a5",

"Energy Balance (Avail. vs Demand)": "#fc8d62",

"Homeostasis Signaling Networks": "#8da0cb",

"Neurodegeneration": "#e78ac3",

"Unclassifiable": "#a6d854"

}

# === 5. Bar chart

cluster_counts = df_included["Thematic Cluster"].value_counts()

cluster_labels = cluster_counts.index

cluster_values = cluster_counts.values

colors = [cluster_colors.get(label, "#999999") for label in cluster_labels]

plt.figure(figsize=(10, 6))

cluster_counts.plot(kind='bar', color=colors, edgecolor='black')

plt.title("Distribution of Articles by Thematic Cluster")

plt.xlabel("Thematic Cluster")

plt.ylabel("Number of Articles")

plt.xticks(rotation=30)

plt.tight_layout()

bar_plot_path = "cluster_bar_plot_colloca_93art.png"

plt.savefig(bar_plot_path)

plt.show()

# === 6. Pie chart

plt.figure(figsize=(8, 8))

plt.pie(cluster_values, labels=cluster_labels, autopct='%1.1f%%', colors=colors, startangle=140)

plt.axis('equal')

plt.title("Proportional Distribution of Articles by Cluster")

plt.tight_layout()

pie_chart_path = "cluster_pie_chart_colloca_93art.png"

plt.savefig(pie_chart_path)

plt.show()

# === 7. Save Excel output

output_dir = r"C:\Users\franc\OneDrive\Desktop\francesco\kefir\sistematic_rev\Asreview_con_meno selezione"

os.makedirs(output_dir, exist_ok=True)

output_file = os.path.join(output_dir, "clustered_articles_Colloca_keywords_93art.xlsx")

df_included.to_excel(output_file, index=False)

print("✅ Excel file saved to:", output_file)

print("📊 Bar chart saved to:", bar_plot_path)

print("🥧 Pie chart saved to:", pie_chart_path)
